# Adult patients’ experiences of NHS specialist services for chronic fatigue syndrome (CFS/ME): a qualitative study in England

**DOI:** 10.1186/s12913-017-2337-6

**Published:** 2017-06-02

**Authors:** Jessica Broughton, Sarah Harris, Lucy Beasant, Esther Crawley, Simon M Collin

**Affiliations:** 1 Department of Psychology, University of Bath, 10 West, Bath, BA2 7AY UK; 2 School of Social & Community Medicine, University of Bristol, Oakfield House, Oakfield Grove, Bristol, BS8 2BN UK

**Keywords:** Chronic fatigue syndrome, Patient experience, NHS England, Qualitative

## Abstract

**Background:**

Few studies have explored patients’ experiences of treatment for CFS/ME. This study aims to fill this gap by capturing the perspective of patients who have been treated by NHS specialist CFS/ME services in England.

**Methods:**

Semi-structured interviews were conducted during the period June–September 2014 with 16 adults who were completing treatment at one of three outpatient NHS specialist CFS/ME services. Interviews were analysed thematically using constant comparison techniques, with particular attention paid to contrasting views.

**Results:**

Three themes were identified: ‘Journey to specialist services’; ‘Things that help or hinder treatment’; and ‘Support systems’. Within these themes nine sub-themes were identified. A wide range of factors was evident in forming participants’ experiences, including personal characteristics such as perseverance and optimism, and service factors such as flexibility and positive, supportive relationships with clinicians. Participants described how specialist services played a unique role, which was related to the contested nature of the condition. Many participants had experienced a lack of validation and medical and social support before attending a specialist service. Patients’ experiences of life before referral, and the concerns that they expressed about being discharged, highlighted the hardship and obstacles which people living with CFS/ME continue to experience in our society.

**Conclusions:**

The experiences of CFS/ME patients in our study showed that NHS specialist CFS/ME services played a vital role in patients’ journeys towards an improved quality of life. This improvement came about through a process which included validation of patients’ experiences, acceptance of change, practical advice and support, and therapeutic outcomes.

**Electronic supplementary material:**

The online version of this article (doi:10.1186/s12913-017-2337-6) contains supplementary material, which is available to authorized users.

## Background

Chronic Fatigue Syndrome (CFS), also known as ‘ME’, is a long-term disabling condition characterised by debilitating fatigue of unknown cause, post-exertional malaise, cognitive dysfunction and disturbed/unrefreshing sleep, plus other symptoms including muscle and joint pain, headaches, and dizziness [1]. CFS/ME imposes an immense burden on patients, carers and families [2, 3]. In the UK, adults who attend NHS specialist CFS/ME services have been ill for a median duration of 3 years, and half of those who were employed at the onset of their illness have ceased working [4].

Around 8000 patients are treated annually by NHS specialist CFS/ME services in England [5]. There are approximately 50 such services in England, many of which were established under the CFS/ME Service Investment Programme (2004–2006) [6]. These services follow guidance provided by the National Institute for Health & Care Excellence (NICE), including specific guidelines for diagnosis, specialist care, and ongoing management, with an overall patient-centred approach to treatment [7].

Qualitative studies in CFS/ME have tended to focus on patients’ experiences of the illness, rather than of care received [8, 9]. Common thematic areas identified by these studies include the high level of functional impairment [10], the specific nature of the fatigue [11], and issues of coping and identity [12, 13], stigma [14], and social isolation [15, 16]. Patients generally reported unmet need in terms of social [17] and medical support [18]. The thread running through these themes is the contested nature of CFS/ME [19, 20]. Although the NHS in England has taken a commendable lead in specialist care provision for adults with CFS/ME, recognition and legitimization of the condition across all levels of the health service remains problematic [21]. An exploration of patients’ experiences of CFS/ME treatments within the NHS is timely and exigent, given opposition from some quarters to evidence-based therapies recommended by NICE and highly discrepant views on the best approaches to treatment [22].

The aim of our study was to explore the experiences of CFS/ME patients who were completing programmes of treatment at three NHS specialist CFS/ME services in England. We used thematic analysis to analyse semi-structured interviews in which we asked questions about the patient journey before, during and at the end of receiving specialist medical care.

## Methods

### Study design

We recruited patients who were concluding treatment at one of three outpatient NHS specialist CFS/ME services in England. These services follow NICE guidelines for diagnosis and management of CFS/ME [7], offering patient-centred programmes which aim to increase patients’ physical, emotional and cognitive capacities, whilst also managing the impact of symptoms. Cognitive Behavioural Therapy (CBT) and Graded Exercise Therapy (GET) are the two main evidence-based therapies, which (or components of which) are used in conjunction with techniques aimed at managing activity, sleep hygiene and relaxation. Treatment programmes typically comprise regular (weekly, fortnightly or monthly) individual and/or group sessions over a period of 3 to 9 months. Patients also receive practical support around employment and the benefits system. Services shared a philosophy of rehabilitation, aiming for ‘recovery’ or ‘significant improvement’, whilst acknowledging that this would not be attained by all patients. A cross-sectional design was adopted with an opportunity sample of 16 patients. Semi-structured interviews were chosen as the most appropriate way to explore patients’ experiences.

### Recruitment

Participants were recruited between July–September 2014. Patients who completed a course of treatment within this period were offered a Patient Information Sheet and ‘Consent to Contact’ Form by a clinician involved in their treatment. Interested patients were required to opt-in to the study by returning the Consent to Contact Form to the research team, after which the principal researcher contacted the patient by telephone to discuss the study in further detail. Participants were not eligible to participate in this study if they were: age < 18 years; too severely affected to be able to participate in interviews; unable to provide informed consent; unable to read and understand the Patient Information Sheet and Consent Forms; or not diagnosed with CFS/ME as a primary diagnosis.

### Data collection and analysis

The potential for the research team to influence findings was carefully considered at all stages of the project [23]. The study adhered to a ‘participatory’ qualitative research paradigm, using an inductive approach which was driven by the data, and which did not hypothesise about potential findings [24]. Researchers were not considered to ‘know best’, and we followed a collaborative approach between the research team and participants [25] in which patients were considered to be ‘experts by experience’ [26]. The semi-structured interview protocol (see Additional file 1 ‘Topic Guide’) was developed through consultation with a Patient Reference Group affiliated with Action for M.E., a UK nationwide CFS/ME charity. Participants were offered the choice of being interviewed in their own home or by telephone. All interviews began with the open question: *“Tell me about your CFS/ME”* and participants were encouraged to guide discussion and introduce their own topics of interest. This led to exploration of some topics not anticipated by researchers. Interviews were audio-recorded, transcribed and analysed thematically in accordance with guidance by Braun and Clarke [27]. Analysis began before completion of data collection in order to ensure that data were as richly detailed and relevant as possible [28]. Techniques of constant comparison informed the analysis and the identification of themes [29]. All transcripts were coded thematically by two researchers [JB and SC], who then used an iterative process (including the use of a thematic map as a visual aid) to agree a final structure of themes and subthemes which best represented participants’ accounts within the remit of the research question.

## Results

### Participants and interviews

The median age of participants was 43 (range 24–62) years, 87.5% (14/16) were female, and the median self-reported duration of illness was 7.5 years (range 1–17) years. The sample was representative of patients treated by the 3 services during 2014 (median age 40 years, 81% (344/424) female), except for a longer duration of illness (median 2 years). Six participants were interviewed face to face in their own homes whilst ten participants were interviewed via telephone. Interview length ranged from 23 min to 57 min, with a mean length of 32 min.

### Themes and sub-themes

Three main themes were identified: (1) ‘Journey to specialist service’ (2); ‘Things that help or hinder treatment’; and (3) ‘Support systems’. Within these themes, 9 sub-themes were identified (Table 1, Additional file 2 ‘Thematic Map’).

**Table 1:** Themes and sub-themes

Theme	Sub-theme
Journey to specialist service	Time taken for referral
	Role of GPs
	Diagnosis and validation
Things that help or hinder treatment	Personal responses
	Acceptance
	Service access and flexibility
Support systems	Mutual support
	Professional support
	Post-discharge support

### Journey to specialist service

#### Time taken for referral

Four participants reported that referral to the specialist service had been a lengthy process, mainly because diagnostic procedures required ruling out other medical conditions. The numerous medical tests and appointments with multiple clinicians was described as being anything from *“hard work”* to *“very very distressing”*. Four participants discussed having procedures, such as scans or tests for brain tumours, which were frightening and *“disconcerting”*. Not having an answer was in itself challenging:

*“I must have been the only patient in the world who was in tears when none of the tests said what was wrong because I just wanted something to sort it out”[P3]*

Participants discussed factors that delayed referral to specialist services for CFS/ME. Some were initially misdiagnosed, for example with depression, multiple sclerosis or glandular fever. Four participants said that ‘watchful waiting’ periods before symptoms were actively investigated were difficult:

*“I was going in every few weeks and they’d ask if it was any better and I was like no not really and it was like okay come back in a few weeks” [P5]*

Some participants had felt the need to *“hide”* symptoms of CFS/ME, particularly from their employer, for example using annual leave to manage symptom flare up for a number of years:

*“I kept it totally secret…and I would just hide on the [pause] at the times I didn’t feel well…I had this secret life” [P6]*

#### Role of GPs

All participants were referred to CFS/ME specialist services by their GPs. Participants reported varied experiences before referral to specialist services. Participants with positive experiences reported that their GPs had been *“very supportive”*, *“brilliant”* and *“fantastic”;* they valued *‘being taken seriously’* and recognised the key role that their GP had played:

*“I said to her I’m really tired I’ve been in bed for ten days and I’m sorry to sound pathetic and she was fantastic because she said no no no we’ll take this seriously” [P7]*

One participant described themselves as *“lucky”* to have a supportive GP, because they were aware from contact with the wider CFS/ME community that other patients had not had good experiences:

*“I went back to my GP who I’m fortunate is very supportive indeed…when I heard about some people’s experiences with their GP I was so grateful for the fantastic GP that I’m lucky to have…because it makes a tremendous difference” [P9]*

Participants with less positive experiences described a number of barriers to accessing specialist services, including a lack of information, having to take a proactive role in asking for diagnostic tests, and GPs’ lack of *“awareness”*, *“knowledge”* or *“belief”* in CFS/ME:

*“My mum did some research and she asked if it was ME and he said that there was no such thing” [P14]*

Two participants discussed their reluctance to be referred to specialist services because of misunderstandings and stigma associated with CFS/ME:

*“When my GP said I’d like to refer you I just laughed…and said, it’s terrible but I did, I said oh isn’t that for people who don’t like work and she really like growled at me in a nice way and said I think you’ve got a lot to learn” [P7]*

#### Diagnosis and validation

Many participants had their CFS/ME diagnosis confirmed when they were assessed by the specialist services. Although some participants described feeling *“relieved”* that diagnosis provided *“an answer”* and *“ruled out”* other conditions, it was a difficult time for the majority. Participants recalled feeling *“angry”*, *“distressed”*, *“frustrated”*, and *“fearful”,* and that the diagnosis represented *“a life sentence”*. The specialist service played a crucial role in supporting patients through this difficult period by providing information and by validating participants’ experiences:

*“it was really nice to feel that um I was being treated by expert professionals who understood the condition and were sympathetic to it and were really committed to helping which was err you know a completely different experience to my GP to be honest” [P2]*

Accepting diagnosis of a contested condition was difficult for some, because of participants’ own negative preconceptions about CFS/ME and the reactions of others. These participants discussed feeling under pressure to *“convince”* or *“prove”* the validity of their experiences:

*“When the doctor suggested as a diagnosis I, um I don’t know…it was like this huge blow and very distressing. The only times I’d heard anyone talk about ME CFS Fibromyalgia it was in very very negative terms, either very sceptical or friends of my mother…who you know she’d talk about being bedridden for years and um how tragic their lives were [laughs] and um I didn’t want to be part of any of those things and…I didn’t see how I could convince other people…I just thought no-one’s going to believe I’m ill…I’m not sure I believe I’m ill” [P6]*

Many participants recalled having a limited understanding of CFS/ME prior to accessing specialist services, having received little or conflicting information about the illness. For many participants, specialist services provided information and explanation of CFS/ME, simultaneously validating and normalising participants’ experiences and symptoms:

*“It was only after I got referred that an explanation was actually given…that it was actually properly described to me and that it was told this is what it is this is what it does to you” [P12]*

### Things that help or hinder treatment

#### Personal responses

Although all participants felt that they had benefited from accessing the specialist service, half recalled finding initial stages of treatment difficult. Many discussed personal responses they believed were key to overcoming challenging periods during treatment. Characteristics described included being *“open”*, “*positive”*, *“proactive”*, *“willing to try anything”*, being able to take *“a leap of faith”,* and having *“perseverance”*.

Participants explained that, particularly during early stages of treatment, advice given by clinicians felt counter-intuitive, and was a departure from the way that symptoms and ‘boom and bust’ cycles had been self-managed prior to accessing services:

*“I said I would rather do something when I can even if it means that I end up in bed for a week afterwards because at least I’ve achieved something…I said I’d rather be asleep and I’ve done something than never do anything” [P14]*

Participants who undertook treatment which involved limiting activity struggled with the restrictive and slow progress that this permitted, describing it as *“disheartening”*, *“painful”* and *“frustrating”*:

*“I was like I don’t want to do baby steps I don’t want to do ten minutes at a time…I wanna be normal and do what I used to do two months ago…so in the beginning it was hard” [P1]*

*“To hear that limitation is very hard… almost can’t breathe sort of overwhelming” [P9]*

#### Acceptance

A second sub-theme highlighted the importance of acceptance in obtaining the most benefit from treatment. Participants discussed a need to accept changes to their lives as a result of developing CFS/ME, and reflected upon what they had lost or relinquished, including social networks, employment, career and study aspirations, and independence:

*“I couldn’t go to university no more because I’d not got my A Levels I couldn’t finish my A Levels at that point I couldn’t work so I couldn’t earn any money at that point…I couldn’t go travelling cause I was too unwell so I literally [pause] everything that I’d planned in my life I couldn’t do-I couldn’t do any of it I had to change change everything and I’m now in a completely different situation to what I had planned” [P12]*

One patient talked about acceptance of their illness in terms of loss and bereavement:

*“You have to go through the grief you have to go through the loss the bereavement the anger the distress of losing so much because you do lose a tremendous amount” [P9]*

The majority of participants recalled having had specific hopes and expectations of referral and treatment, including to confirm diagnosis, “*reduce symptoms”,* obtain *“answers”,* and manage symptoms better. The most frequently discussed hope prior to accessing specialist services was for a *“cure”*, a return to previous health or abilities, or to *“get back to what I was used to”*:

*“My expectations were quite high when I started thinking it’s going to sort me out, they’ve found out what I’ve got now it’s going to sort it out, but it’s not that way” [P8]*

Participants described a *“big”* or *“steep learning curve”* during early stages of treatment, highlighting the importance of being *“willing to change”* and being prepared to *“say goodbye to your old life completely”* in order to engage fully with treatment. Five participants discussed needing to overcome initial scepticism about the likelihood of treatment success:

*“I just remember looking at (clinician) and looking at my husband and just going oh whatever” [P1]*

Some participants attributed their early hopes and expectations to lack of knowledge about CFS/ME and to being unsure about what to expect from a specialist service. The information about CFS/ME provided by the specialist clinics was valued by many, but focus on management remained difficult whilst hopes for a cure remained:

*“It’s just frustrating because nothing makes it better I suppose…it was good getting the information but it doesn’t get rid of it” [P14]*

Time appeared to influence acceptance, with some participants recalling a gradual acceptance that treatment might not be curative:

*“Obviously you know there isn’t some kind of magic potion or anything that helps you get better…but managing your ME and managing the kind of boom-bust that they talked about and putting in kind of regular resting breaks or Mindfulness or you know whatever’s helpful, I think those kind of things are the best way of-of supporting yourself so that you can function normally and get- you know get through life as normally as possible” [P10]*

Various approaches were used by clinicians to assist with setting goals, including addressing expectations of cure at the patient’s first appointment, delaying discussion until some progress had been made, providing information advising participants of the long term nature of CFS/ME, and being *“open”* and *“honest”* about the limitations of treatment. Moving away from the idea of cure towards goals related to management of CFS/ME was linked to obtaining information and acquiring knowledge about the condition:

*“there’s always a silver lining…I would argue that I live life fully in the slow lane now, and that’s quite a nice way to be…and I know if I go out into the fast lane there will be consequences, but I can make that choice sometimes I will choose to go out into the fast lane to do something but then I know it’s, you know there might be a relapse or, or I’ll have to have a couple of days just not doing much at all…but that’s management” [P9]*

Participants recalled that clinicians also assisted with and encouraged the development of new goals which had not been held prior to accessing specialist services. Some viewed these as vital to treatment success, representing a shift in focus towards management rather than cure. Changing goals was recognised as a *“slow”* and challenging process. New goals were described as *“smaller”, “a lot more realistic”* and *“more sensible”*, involving the *“breaking down”* of existing goals, lowering expectations and focusing on the *“day to day”* rather than the future:

*“[things] that once seemed the small things perhaps become the big things…it’s having to change your whole mind-set really which most of us don’t do unless we’re pushed into a crisis but it didn’t happen overnight” [P13]*

#### Service access and flexibility

Participants discussed accessibility in terms of being able to attend appointments and accommodate treatment programmes around other commitments. The majority of participants were pleased with the practical accessibility of clinics, describing journeys as being *“manageable”* or *“easy”*. However, all participants mentioned that accessibility could be a barrier to attendance. Whilst all reported that ease of access to the clinic improved over time as symptoms improved, travel during the early stages could be *“incredibly hard”,* with participants finding the journey *“stressful”* and *“quite troublesome”*, and needing to recover after attending appointments:

*“It was quite difficult because it’s 40 minutes from home so it was a really tiring journey…made it quite a long day…I was usually asleep for a couple of days afterwards” [P14]*

Some discussed the importance of good public transport links to the specialist service, whilst others felt that they would not have been able to attend appointments without use of a car. Some participants discussed a need for assistance to attend appointments, including help from partners or friends, particularly when symptoms were severe:

*“In the early days we were able to arrange appointments around [my husband’s] diary so he was able to take me…‘cause I didn’t feel I was able to manage the bus” [P16]*

Others said that work commitments could be a barrier to attending appointments, one patient describing how it had been a struggle to arrange time off work to attend the specialist service:

*“my appointments were generally on days I didn’t work and the two day thing I managed to get, finally, because I was outed as, you know, they put me down as a disabled employee, which you know I didn’t want, but it allowed me to get this time” [P6]*

Participants noted that accessing the clinic would have been difficult if experiencing severe symptoms, and concerns were raised about the ability of those severely affected by CFS/ME to access specialist services:

*“The severely affected people with ME…a forgotten group of people who are invisible and that’s a group of people who really really need that help, which they’re not getting…because they wouldn’t be able to get to it” [P9]*

Flexibility in the frequency and mode of appointments was valued by participants, with two participants saying that they appreciated being offered later appointments because of travel burden and symptom fluctuation:

*“In the beginning it was quite hard but they were always really good ‘cause I used to struggle in the mornings the mornings used to be my hardest time so they were really good at giving me afternoon appointments” [P1]*

The option to have some appointments by telephone was highly valued, particularly when symptom severity or travel problems made attendance difficult:

*“I remember ringing up to cancel because I couldn’t get a lift and they suggested [a telephone appointment] I hadn’t realised we could do that but it was really really useful” [P16]*

Two participants mentioned Skype as a possibility. One had used Skype as part of a clinical trial and felt that it provided *“an improvement on a phone call”*, but that it would not be appropriate to replace all face to face sessions with Skype.

### Support systems

The third theme identified during analysis related to systems of support that participants felt were an important aspect of their experiences at the specialist services. Within this theme we identified three sub-themes; ‘Mutual support’, ‘Professional support’ and ‘Post-discharge support’.

#### Mutual support

Nine participants attended only one-to-one treatment sessions with clinicians at the specialist services, and seven attended a mix of individual and group sessions. Despite generally strong positive recollections of group experiences, many participants had initially held negative perceptions about what groups would entail, leading participants to feel reluctant or *“dubious”* about attending group sessions. Participants recalled worrying about groups being *“touchy-feely”*, pressure to disclose *“things that are more personal that maybe you wouldn’t want to share in a group”*, that group members might *“bring each other down”* or *“take on other people’s traumas”*, and not wanting to *“hang out with a bunch of sick people”*:

*“At the time I had this vision of just sitting in a room with people and we would all be talking about our ailments and I thought that’s not gonna help me” [P16]*

One participant had wanted to access one-to-one sessions but was unable to do so because of a long waiting list:

*“I don’t really like do lots of social situations and group things but I was told in no uncertain terms that it’s either the group thing or you’ll wait years and years for individual ones so I thought okay I’ll go for the group one” [P15]*

The majority of participants recalled group sessions positively, with benefits including: relating to other patients; the opportunity to share experiences and *“stories”*; receiving support from group members; supporting others; hearing about others’ experiences; and having their own personal experiences and symptoms validated and normalised:

*“Listening to other people’s stories and realising um that other people were experiencing exactly the same thing and I wasn’t imagining it and it wasn’t all in my head and I wasn’t being weak I wasn’t being pathetic I wasn’t being lazy” [P7]*

Some participants thought it important that a group” *clicked”* or *“gelled”* in order for the benefits of mutual support to be realised. In particular, use of humour and having *“things in common”* were recalled as facilitators of group bonding:

*“I went into a room with about a dozen other people…all different ages backgrounds sort of thing…and then we went round and shared our stories…and it was quite an eye-opener really how different we all were and how the illness affected us each differently but we were all there with this common condition” [P11]*

#### Professional support

Relationships with clinical staff working for the CFS/ME service were highly valued by participants, particularly those accessing one-to-one treatment sessions. Many participants had positive experiences and comments about the clinicians they had worked with. Staff were described as *“patient”*, *“compassionate”*, *“reassuring”*, *“knowledgeable”*, *“inspiring”*, *“friendly”*, and *“helpful”*, ensuring that participants felt *“comfortable”*, *“at ease”*, *“valued”*, *“supported”* and treated *“as an equal and an individual”*. Participants described feeling able to be unguarded, and discussed the value and importance of being believed:

*“I could relax and I could tell her how I felt without feeling like she’s going to dismiss me and I could you know…use humour if I wanted I could swear if I wanted…I realised I didn’t have to worry she was thinking aha you know like looking into my mind to see what was really going on” [P6]*

Two participants contrasted their positive experience with clinicians at the specialist service with negative previous healthcare experiences:

*“I’ve seen a lot of different doctors and things and because they don’t understand the condition it doesn’t really feel like they care very much and they’ve got other patients to deal with and they don’t have time…so it’s really refreshing to meet someone who understands it and who really cares you know they really believe what you experience some people think it’s still in your head and things and it’s a bit patronising” [P10]*

#### Post-discharge support

For some participants, completion of a treatment programme meant the end of contact with the service. Some discussed this positively, with one participant *“feeling ready”* and another, who had *“significantly improved”*, feeling *“happy”*. One participant felt that it was a natural progression – “*you’ve kind of flown the nest [laughs] you’re being set free”* – and another said that they’d had *“a pretty good bite of the cherry really”*. However, the majority of participants (10/16) were worried about their ability to cope following discharge, and were concerned about the level of support that would be available:

*“The biggest thing I’m missing and I’m sure a lot of people would say the same is just having um some support after the course” [P10]*

Some participants had been advised that they could contact the service by telephone should they require further support or re-referral:

*“I’ve just been discharged but there’s a safety net in that they said we don’t just drop you, if you need help or if you’re struggling then don’t be a stranger just pick up the phone” [P13]*

Others had been offered a review appointment 6–12 months after completion of treatment, although this follow-up did not assuage concerns about life after discharge:

*“Like there isn’t any [aftercare] you do your ten sessions and that’s it…you can speak to somebody within six months but you just get to speak to somebody and that’s it you can ask to be referred again but the waiting lists are that big considering you’ve done it once I don’t know whether or not you’d get to do it again” [P15]*

Specialist services were seen to provide *“reassurance”*, “*backup”* and were viewed as a *“security blanket”*, *“safety blanket”* or “*lifeboat”*. Some participants said that they would prefer to err on the side of caution and retain links with the service:

*“The occupational therapist asked me how I was feeling and I said the same thing you know the mixed emotions thing and she sort of asked well what would make you feel better and I was like oh I don’t know…maybe knowing that if I needed to come back I could” [P1]*

Some felt that they would miss the routine and structure of attending the service – *“quite a regular part of my week”* – or would struggle to maintain progress independently:

*“That would be really nice if we could like have a regular kind of meet up…just to check in and see how you’re doing what your progress is if you need any support or things like that…because otherwise you feel like you learn these tools and then you’re kind of left out in the open a bit” [P10]*

Strong negative reactions to discharge were expressed by several participants in the context of their experiences before attending the specialist service, when they had felt unsupported by GPs, family, friends, colleagues and employers, and had experienced stigma and lack of understanding:

*“When the specialist discharged me I uh I’m not blaming him but I was left feeling very alone-the illness itself makes one quite um [pause] lonely because uh social activity’s quite tiring” [P11]*

These strong reactions included fears about relapse and being *“abandoned”,* a sense of “panic” and even *“bereavement”*:

*“It’s like you’re having another bereavement cause like this thing has changed your life…it would have been better if you could maybe have known you could go up there for maybe like top-ups once a month or once every 3 months just so you wasn’t like you’ve had those sessions you’re fixed goodbye” [P15]*

Some of the mutual support systems developed between patients were maintained post-discharge. Participants from one of the specialist services discussed arranging their own meetings independently from the clinic following completion of treatment:

*“Our group meet every couple of months so we’re all supporting each other really” [P8]*

## Discussion

This is the first study to provide an insight into the experiences of adult patients who have attended NHS specialist services for CFS/ME. Our study reiterates the challenges faced by CFS/ME patients, and reveals the important and unique role that specialist services can play in the lives of people living with CFS/ME. Participants described lengthy and often difficult journeys to reaching a specialist service, and reflected on the challenges posed by the contested nature of CFS/ME. Convoluted diagnostic pathways and lack of validation were particularly challenging obstacles en route to referral, compounded by lack of social and professional support.

NHS specialist CFS/ME services have evolved to deliver programmes of treatment that enable patients to embark on a process of rehabilitation. The services in our study shared a philosophy of aiming for recovery or ‘significant improvement’, whilst acknowledging that this would not be attained by all patients, that ‘recovery’ would mean different things to different people, and that talking about a patient’s pre-illness lifestyle could be part of the therapeutic process. The process demands effort and commitment from patients, but the experiences of patients in our study demonstrate the potential for improving patients’ quality of life. This has been demonstrated quantitatively [30], but our study illustrates what changes in numeric questionnaire scores actually mean for patients, and what patients have to go through to achieve that improvement.

All participants reported having benefited in some way from accessing specialist services, and their accounts highlighted factors which appear to mediate the success of treatment programmes. These factors include participants’ own reactions and attitudes towards treatment, which were deemed crucial in fostering engagement and perseverance during the difficult early stages of treatment. Many discussed the importance of acceptance and the use of goal setting to navigate adaptations to a life with chronic illness. The role played by clinicians at the specialist services was recognised and highly valued by participants.

Discussion of participants’ reactions to discharge identified the specialist services as a source of long-term support for participants, with participants’ realism and optimism for the future being tinged with fears about disconnecting from services. These concerns were particularly acute for those patients who had encountered the greatest difficulties in obtaining specialist treatment.

### Our findings in the context of previous studies

We don’t have figures for the proportion of the ‘clinical iceberg' visible in secondary care, but we assume that not all adults with CFS/ME access specialist services. Most areas of England have geographical access to a specialist service [5], but it is evident from the experiences of patients in our study that barriers to access remain. Without conducting a comparative study, we don’t know to what extent the participants in our study might be considered ‘the lucky ones’, i.e. people who have accessed a specialist service and completed a programme of treatment. Nonetheless, there is a reasonably rich seam of qualitative literature around patients’ and health care professionals’ experiences of CFS/ME, if not of treatment per se.

#### Journey to specialist service

One part of the patient journey emerged as a main theme in adult patients’ experiences of services, namely; the long and often tortuous path through primary care, beginning with the patient’s own gradual recognition that something was *“just not right”*, through inconclusive and negative tests, misdiagnoses, and lack of information, before finally being referred to a specialist service. This long and difficult journey to specialist care has been described by parents of paediatric CFS/ME patients [31]. NICE guidance states that *“*Referral to specialist CFS/ME care should be offered: within 6 months of presentation to people with mild CFS/ME; within 3-4 months of presentation to people with moderate CFS/ME symptoms; immediately to people with severe CFS/ME symptoms*”* [7]. The median duration of illness in our sample was 8 years, with a range of one to 17 years.

Our study showed that negative experiences early in the patient journey can arise partly from patients’ own perceptions of CFS/ME. Although these are likely to reflect societal attitudes to CFS/ME, thereby posing a somewhat intractable problem, GPs and other primary health care professionals clearly have an important role to play in addressing delegitimization and misinformation, and in improving the standard of care. This was evident in the positive experiences of those patients in our study who were believed and supported by well-informed GPs. Being diagnosed with CFS/ME triggering a range of emotions in patients. Practitioners in specialist services might be best-placed to support patients at this stage in their journey [21], but the key elements in providing that support – information, explanation, validation, empathy – are within the remit of patient-centred primary care.

#### Things that help or hinder treatment

Of the underlying themes relating to entry into and subsequent engagement with treatment, perhaps the most fundamental was ‘acceptance’. Patients’ acceptance of changes, to their lifestyle and identity, was particularly important in helping patients negotiate the difficult early stages of treatment. Acceptance and identity are recurring central themes in qualitative studies of CFS/ME [8, 9, 12, 13]. These are themes common to chronic illness, but certain aspects of CFS/ME – the long journey to diagnosis, the contested nature of that diagnosis [19, 20], the responses of society [17] and significant others [16, 32], varied symptomatology [1], unknown aetiology, and broad age range of those affected [33] – present particular challenges to patients and clinicians.

Patients’ views about personal responses that are conducive to overcoming challenges during treatment can be loosely mapped to known predictors and mediators of treatment outcomes. These include acceptance and neuroticism [34], the acceptability of expressing emotions [35], fear and embarrassment avoidance [36, 37], all-or-nothing behaviour [37], beliefs around activity and inactivity [10], sense of control over symptoms [38, 39], and ‘psychological flexibility’ [40]. Ideally, a patient-centred approach to treatment will accommodate diverse personal responses and behaviours.

The goal attainment approach used by services, and commented upon as one of the more challenging aspects of treatment, has been associated with improvement in quality of life in CFS/ME patients regardless of initial health status [41]. The effectiveness of this approach, in conjunction with other factors such as services’ attention to detail around issues such as accessibility and flexibility, would contribute to favourable views of services among patients. ‘Alternative’ approaches to treatment, such as accepting the legitimacy of patients’ accounts of their illness and providing realistic expectations of treatment outcomes [42], have already been adopted, and our study confirms that referral to a specialist service is highly valued [43].

#### Support systems

The therapeutic relationship, encapsulated in the ‘Professional support’ subtheme, will clearly be of importance in predicting treatment outcomes, with one study in CFS/ME treatment indicating ‘positive outcome expectations’ and ‘task agreement’ as being important during the early stages [44]. One element of treatment which was particularly valued by participants was normalisation of experience through attending group based treatment programmes. This reflects the legitimisation and validation which appeared key to participants’ encounters with others in group therapy [45]. It was clear that group sessions, despite initial reluctance and wariness, began to offset individual experiences of isolation prior to referral.

### Strengths and limitations

Our study strikes a balance in capturing differences and similarities between patients in terms of their experiences of NHS specialist CFS/ME services. The participants in our study were recruited from specialist services in three quite different settings – London, the south west and the north west of England – but these services can be considered broadly representative of the larger (>250 patients per year) specialist services in England. Similarly, all participants were diagnosed and treated in accordance with UK NICE guidelines [7], but between-service variation in the structure and content of treatment programmes, and the patient-centred ethos of the guidelines, means that the patients in our study did not all receive exactly the same course of treatment. We did not seek to explore patients’ experiences of specific treatments or components of treatment programmes such as CBT and GET.

The size of our opportunity sample was constrained by resources, and was at the lower end of what might be considered sufficient to obtain saturation in all topics [46]. Interview length was constrained partly by the time available to conduct the study, but we were also mindful of not wanting to over-exert patients who may still be having to self-manage their activities, including cognitive exertion. The topic guide was arrived at through consultation with the Action for M.E. patient reference group, which gave us some reassurance that we were able to cover the main topics within the allotted time. Although we captured a range of patient experiences in a sample which was heterogeneous and broadly representative of patients who access NHS specialist CFS/ME services in terms of age and sex, the patients in our sample reported longer durations of illness. Patients who are ill for a shorter period of time before referral may experience specialist services quite differently, particularly in relation to sub-themes such as ‘Time taken for referral’ and ‘Acceptance’. Our sample was too small to explore patients’ experiences of specialist services in relation to the ME/ME, or in the presence of comorbidities such as depression and fibromyalgia [1]. Our remit to explore the experiences of the ‘majority’ of CFS/ME patients meant that we excluded severely affected patients. These patients merit special research attention, given the severity of their illness [47, 48] and specific issues such as lack of domiciliary care provision [49]. These sampling issues are likely to restrict the generalizability of our findings to all CFS/ME patients. Similarly, patients attending smaller CFS/ME services may have different experiences from those who participated in our study.

The other principal limitation of our study is that we recruited patients who had reached the end of treatment. This means that the positive experiences that we have reported could be considered to represent ‘success stories’. We do not have figures for drop-out rates, which might reflect dissatisfaction with services and/or competing health issues. We would propose interviews with patients who do not complete treatment as an important area for future qualitative research. Our findings are susceptible to selection bias if patients were motivated to participate by a sense of gratitude and an altruistic desire to ‘give back’ to the clinic after a positive experience. We know that black and minority ethnic groups are under-represented among patients attending specialist CFS/ME services [17, 50], and people living with CFS/ME in these population groups may have different or particular experiences [51]. Finally, the long-term course of CFS/ME after treatment is not well-characterized, either quantitatively or qualitatively. We would need to repeat our study with a sample of patients several years post-treatment to explore whether positive outcomes at the end of treatment translate into recovery or sustained improvement in quality of life. Finally, alternative methods of analysis, such as Interpretive Phenomenological Analysis, may have given a different focus to our analysis. We trust that our thematic approach will create a framework for further qualitative research within this area.

### Implications for health services

Although knowledge and awareness of CFS/ME in primary care has improved over the past decade [21], it is clear from our study that some patients still have negative experiences. Some of these stem from uninformed health care professionals, and lack of confidence that non-specialists have in making a diagnosis [52, 53]. These problems are not unique to the UK [18, 54], and initiatives based on better provision of information for GPs [55, 56], changes to medical training [57] and ‘early intervention’ [58] could be applied across health systems.

Patients’ pre-treatment experiences gave rise to strong feelings about being discharged, with many fearing a future in which they were no longer connected to the service. Although some services addressed this by offering review appointments and telephone contact, long-term support is one aspect of service provision which is not covered by current guidelines. One of the services provided ‘Moving Forward’ and ‘Relapse Prevention’ plans towards the end of treatment. The Moving Forward plan was based on questions such as “What are my next steps?”, “How will I achieve these?”, “What may get in the way?”, and “How will I deal with them?” The Relapse Prevention plan comprised a ‘CFS/ME Toolkit’ (helpful strategies learnt in sessions), ‘Relapse Signature’ (early signs of relapse), and ‘Relapse Drill’ (what do I need to do when I experience signs of relapse). Some patients had continued to meet in informal self-support groups, an arrangement which might benefit from formal professional support [59]. Our study showed the benefit to patients of professional and social support obtained via the specialist services, beyond clinical care. This suggests that services need to be resourced to provide long-term support for patients, and to be able to focus on long-term pragmatic outcomes such as return to employment [60]. Novel means of delivering care, such as graded exercise via teletherapy [61], also need to be tested.

## Conclusions

The experiences of CFS/ME patients in our study showed that NHS specialist CFS/ME services had a vital role to play in patients’ journeys towards an improved quality of life. This improvement came about through patients’ acceptance of their illness, therapeutic outcomes, and practical advice and support. Patients’ experiences of life before referral, and the concerns that they expressed about being discharged, highlighted the hardship and obstacles which people living with CFS/ME continue to experience in our society.

## Additional files

Additional file 1: Topic Guide: semi-structured interview protocol. (DOCX 16 kb)

Additional file 2: Thematic Map: figure illustrating themes and subthemes arising from thematic analysis of interview transcripts. (TIFF 279 kb)
