# Supplementary material for: Adult patients’ experiences of NHS specialist services for chronic fatigue syndrome (CFS/ME): a qualitative study in England
Source: BMC Health Serv Res. 2017 Jun 2;17:384. doi: 10.1186/s12913-017-2337-6 (PMC5457632; doi:10.1186/s12913-017-2337-6)
Supplement: Supplementary file 1 — Topic Guide: semi-structured interview protocol. (DOCX 16 kb) [file 12913_2017_2337_MOESM1_ESM.docx]

**What are patients’ experiences of NHS Specialist treatment for CFS/ME?**

**Topic Guide.**

Welcome, introduction, stress confidentiality. Discuss consent, sign form or check continues to be happy with consent.

1. **Tell me about your CFS/ME?**

Prompts: How long have you had it? Have you tried other treatments or therapies? What treatment were you receiving before referral to [specialist service]?

1. **Tell me about your current treatment?**

Prompts: What current treatment involves? What is it called? How often do you access it? Who delivers this treatment to you (i.e. types of healthcare professionals)?

1. **How accessible is the specialist service you currently attend?**

Prompts: How often do you attend for treatment? How do you travel to the specialist service? Does attendance have health consequences (i.e. post exertional malaise) which is more significant than what you normally experience?

1. **What was it like to be referred to specialist treatment?**

Prompts: Was this a positive or negative thing for you? How was the news delivered? By whom?

1. **What had you wanted from specialist treatment prior to accessing services?**

Prompts: Do you have goals for specialist treatment? Did / do you expect specialist treatment to differ from your previous treatment? In what way?

1. **Do you think accessing a specialist service has made a difference?**

Prompts: Are things better or worse now? Which aspects of specialist treatment are most / least beneficial to you?

1. **How would you describe your relationship with staff at the specialist service?**

Prompts: Do you have rapport with staff? Do you feel that they listen to you / your views are taken into account? Can you rely on staff if you need? Does your relationship with staff have any impact on your experience of treatment?

1. **Are there other things that would be helpful in terms of specialist treatment for CFS/ME?**

Prompts: Are there aspects of your CFS/ME which are not helped/addressed by the specialist treatment you are currently accessing? How important is this?

1. **Are you accessing psychological services as part of your treatment?**

Prompts: If so, how do you feel about this? Had you accessed psychological services prior to referral to specialist services for CFS/ME?

1. **Has accessing psychological services made a difference to you? (positive or negative)**

Prompts: Has psychology helped with the physical symptoms of your CFS/ME? Are there other benefits to accessing psychological services?

1. **What is it like to access psychological treatment for a condition with physical symptoms?**

Prompts: Is it important what type of treatment you receive? Why / Why not?

1. **Do you have anything else you’d like to add?**

Prompts: Was there anything we should have asked, but didn’t?
