# Supplementary figures and images for: Adult patients’ experiences of NHS specialist services for chronic fatigue syndrome (CFS/ME): a qualitative study in England
Source: BMC Health Serv Res. 2017 Jun 2;17:384. doi: 10.1186/s12913-017-2337-6 (PMC5457632; doi:10.1186/s12913-017-2337-6)

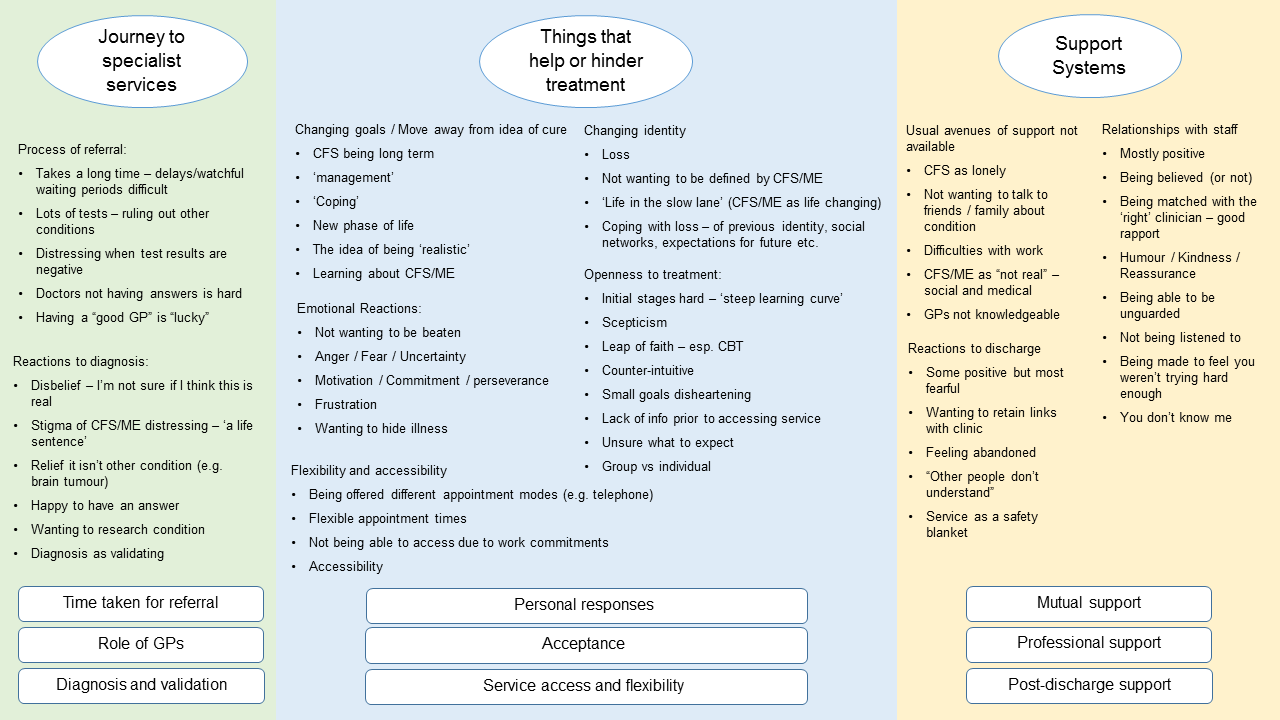

Supplement: Supplementary file 2 — Thematic Map: figure illustrating themes and subthemes arising from thematic analysis of interview transcripts. (TIFF 279 kb) [file 12913_2017_2337_MOESM2_ESM.tif]
